# Maternal microRNAs linked to birth deficits due to prenatal alcohol exposure are dominant modifiers of gene expression in invasive trophoblast cells

**DOI:** 10.3389/fcell.2026.1834760

**Published:** 2026-05-28

**Authors:** Deepa Upreti, Christina D. Chambers, Rajesh C. Miranda

**Affiliations:** 1 Department of Neuroscience and Experimental Therapeutics, Naresh K. Vashisht School of Medicine, Texas A&M University, Bryan, TX, United States; 2 Department of Pediatrics, Division of Environmental Science and Health, UC San Diego, San Diego, CA, United States

**Keywords:** fetal growth restriction, miRNAs, placenta, prenatal alcohol exposure, transcriptional regulation

## Abstract

**Introduction:**

Prenatal alcohol exposure (PAE) can impair placental development and result in fetal growth restriction. However, the underlying molecular mechanisms are unclear. We previously found that PAE in some, but not all pregnancies, resulted in significantly elevated circulating levels of 11 microRNAs (HEamiRNAs) in the 2nd trimester. This subgroup of PAE women experienced adverse infant birth outcomes due to PAE, Subsequently, these HEamiRNAs collectively but not individually, were found to inhibit a defined set of placental epithelial-mesenchymal transition (EMT) genes and, inhibited invasiveness of placental trophoblast cells.

**Methods:**

Here we extend these findings by performing whole-transcriptome analysis to dissect the contribution of ethanol compared to HEamiRNAs in HTR-8/SVneo cells, a model for invasive, human extravillous trophoblasts.

**Results:**

We observed a large, distinct and statistically greater effect of HEamiRNAs mimics on trophoblast transcript profiles irrespective of ethanol exposure, specifically on EMT, extracellular matrix, angiogenesis and immune signaling pathways among others. Weighted gene co-expression network analysis (WGCNA) identified additional placental growth networks related to epithelial cell proliferation and differentiation and MAPK/PI3K, Hippo and Rap1 signaling.

**Discussion:**

This study provides a mechanistic framework to explain that the effects of PAE vary across pregnancies, because they are contingent in part, on the elevation key maternal circulating miRNAs.

## 1 Introduction

Prenatal alcohol exposure (PAE) can compromise key developmental processes throughout all stages of gestation, decreasing placental function, impairing fetal growth and development, disrupting brain maturation and leading to long-term neurobehavioral impairment (Petrelli et al., 2018). Alcohol consumption is common among individuals of reproductive age, and the global prevalence of alcohol use during pregnancy was estimated to be 9.8% (Popova et al., 2017). State-wide biochemical assessments in newborns indicate the presence of PAE in 8%–15% of all pregnancies in the general North American population, at least during the last trimester (Bakhireva et al., 2017; DiBattista et al., 2022; Umer et al., 2020). A recent prospective case ascertainment study in four elementary school systems across the US suggested that Fetal Alcohol Spectrum Disorders (FASDs) are present in 1%–5% of the general population (May et al., 2018). Alcohol readily crosses the placenta and can interfere with early developmental processes, including placental formation and fetal growth (Ohira et al., 2019). However, for reasons that are not well understood, pregnancies with similar levels of PAE can result in varying severity of outcomes, and not all exposed pregnancies result in cardinal craniofacial anomalies and growth deficits associated with FASD. Our previous studies suggested that microRNAs (miRNAs), small, non-coding RNA molecules that regulate gene expression post-transcriptionally, primarily by binding to the 3′untranslated regions of target mRNAs (Upreti et al., 2025), may explain birth outcome variations following PAE.

In a longitudinal study of alcohol and pregnancy outcomes (Balaraman et al., 2016), we previously reported that some pregnant women with heavy prenatal exposure to alcohol exhibited elevated circulating levels of 11 microRNAs (miRNAs, hsa-miR-222-5p, hsa-miR-187-5p, hsa-miR-299-3p, hsa-miR-491-3p, hsa-miR-885-3p, hsa-miR-518f-3p, hsa-miR-760, hsa-miR-671-5p, hsa-miR-449a, hsa-miR-204-5p, and hsa-miR-519a-3p) in blood plasma during the second trimester of pregnancy and that women with this plasma miRNA signature (termed _HEa_miRNAs) subsequently gave birth to infants with growth and neurobehavioral deficits. In contrast, women with heavy PAE who did not exhibit this miRNA signature, proceeded to have unremarkable birth outcomes, as did non-alcohol-exposed women. Moreover, levels of circulating _HEa_miRNAs during the 2nd trimester explained between 24% and 31% of the variance in morphometric estimates of growth in new-born infants (Tseng et al., 2019b). The cellular source of _HEa_miRNAs remains unclear. Our previous assessment (Tseng et al., 2019b) suggests that the placenta itself is at least one major source, though other tissues may also contribute to this secreted miRNA pool. We also previously showed in a cross-species, rodent to non-human primate comparison, that _HEa_miRNAs collectively but importantly, not individually, statistically moderated the effects of ethanol on placental epithelial-mesenchymal transition (EMT) genes. Further studies in human trophoblast cells, showed that _HEa_miRNAs collectively but again, not individually, decreased cell cycle progression, inhibited invasiveness, and inhibited EMT specifically in placental trophoblasts. *In vivo*, tail-vein delivery of 8 murine-conserved _HEa_miRNAs to pregnant mice resulted in placental and fetal growth deficits (Tseng et al., 2019b). A key finding of our previous studies was that no single _HEa_miRNA recapitulated the effect of the entire pool. Adverse effects on placental growth were only achieved by the collective action of this miRNA pool. Moreover, while previous *in vitro* study using HTR8 cells focused on specific EMT- and invasion-related gene targets, the current study uses whole-transcriptome and network-level analyses to define broader gene networks and programs regulated by _HEa_miRNAs. Accordingly, we used a pooled set of 11 miRNAs (_HEa_miRNAs) to assess their combined biological effects on the transcriptome, rather than testing individual miRNAs.

Unexpectedly, bulk transcriptomic analysis of mouse placenta (mixed labyrinthine and junctional zone tissues), following maternal tail-vein injection of the above 8 murine-conserved _HEa_miRNAs, showed that activators of Notch signaling were significantly enriched by these miRNAs (Pinson et al., 2023). Though in that study, *post hoc* deconvolution strategies were not effective in identifying the impacted cell-types, the Notch signaling pathway is an established activator of EMT (e.g. (Kostina et al., 2016; Zhou et al., 2016)), and therefore this outcome was contrary to our previous observations that collectively _HEa_miRNAs were EMT inhibitors in trophoblast cells. Such outcomes, contrary to predictions, suggest the presence of compensatory biological pathways that may serve to mitigate effects of PAE. Therefore, in the current study, we conducted a new whole-transcriptome analysis to assess the collective effects of the 11 identified human _HEa_miRNAs specifically on HTR-8 cells, a model for invasive, human extravillous trophoblast cells (EVTs) that invade the uterine endometrium and remodel maternal spiral arteries (Davies et al., 2016). Moreover, we assessed the effects of sequential exposure to ethanol and _HEa_miRNAs, since, in our initial studies in pregnant women, PAE preceded the observed elevation in _HEa_miRNAs, and a majority of pregnant women curtailed alcohol consumption following pregnancy awareness (Balaraman et al., 2016). We asked whether sequential exposures to ethanol and _HEa_miRNAs could result in differential regulation of gene expression. Our analysis focused on genes involved in EMT, angiogenesis, inflammation, and broader cellular processes including metabolism, proliferation, and stress response. Using differential expression analysis and weighted gene co-expression network analysis (WGCNA), we identified coordinated gene networks and key regulatory pathways affected by these exposures. We report that the effects of _HEa_miRNAs significantly outweigh the effects of alcohol alone. This work provides mechanistic insight into how maternal circulating miRNAs and ethanol may cooperatively disrupt trophoblast function, contributing to variability in placental dysfunction and fetal growth restriction.

## 2 Materials and methods

### 2.1 Cell lines and culture conditions

All the reagents were purchased from Thermo Fisher Scientific unless otherwise specified. The HTR-8/SVneo extravillous trophoblast cell line was obtained from ATCC (Cat No. CRL-3271) and maintained in RPMI-1640 medium supplemented with penicillin (100 U/mL), streptomycin (100 μg/mL), and 5% vol/vol FCS. Cells were maintained at 37 °C in a humidified atmosphere containing 5% CO_2_. Culture medium was replenished every 2–3 days, and cells were passaged every 4–5 days. All experiments were performed using cells at passage numbers below 10 to ensure experimental consistency.

### 2.2 Cell death and apoptosis analysis

To assess the cytotoxic effects of ethanol exposure, HTR-8/SVneo cells at a density of 7x10^3^ cells/well were seeded in 96-well plates. The cells were subjected to one of four ethanol treatments: 0 mg/dL, 60 mg/dL (13 mM), 120 mg/dL (26 mM), or 320 mg/dL (70 mM) for either 24 h or 48 h. These concentrations were selected to represent a range of physiologically relevant exposures, including levels associated with moderate to heavy alcohol use that can be attained in human populations (Adachi et al., 1991). Cytotoxicity was measured using the CyQUANT™ LDH Cytotoxicity Assay Kit (Thermo Fisher, Cat. No. C20301), following the manufacturer’s instructions and quantified on a microplate reader (Tecan Spark). Apoptotic cell death was assessed using the Caspase-Glo® 3/7 Assay System (Promega, Cat. No. G8091), and luminescence quantified using a multimode plate reader (Agilent BioTek Synergy 2).

### 2.3 Ethanol exposure and miRNA transfection on trophoblast cells

HTR-8/SVneo cells were seeded in 6-well plates at a density of 7.5 × 10^4^ cells/well and cultured overnight. Cells were divided into two ethanol exposure conditions: (1) pre-ethanol, where ethanol was added at the time of cell seeding, and (2) post-ethanol, where ethanol was added after transfection. Transfections were performed using Lipofectamine RNAiMAX Transfection Reagent (Thermo Fisher, Cat. No. 13778) and 11 miRIDIAN® miRNA mimics (Dharmacon) corresponding to previously identified _HEa_miRNAs: hsa-miR-222-5p, -187-5p, -299-3p, -491-3p, -885-3p, −518f-3p, −760, -671-5p, −449a, -204-5p, and −519a-3p (Tseng et al., 2019b). A mixture of these miRNAs was prepared at equimolar concentrations as described previously (Tseng et al., 2019b). A non-targeting scrambled control (Dharmacon, Cat. No. CN-001000-01-05) was used in parallel. After 5–6 h of the transfection, media were replaced with Opti-MEM containing ethanol at final concentrations of 320 mg/dL. We used a dose of 320 mg/dL for transcriptomic analyses, as it did not induce cytotoxicity but provided a robust experimental exposure condition that is documented to be attained in adults with alcohol use disorders (Adachi et al., 1991). Ethanol exposed cultures were maintained for 48 h in sealed containers placed in dedicated incubators to minimize evaporation. At 24 and 48 h, culture media were collected to confirm ethanol concentrations using gas chromatography as we previously published (Chung et al., 2022; Pinson et al., 2022; Tseng et al., 2019a). On day 4, cells were harvested by gentle trypsinization, washed with RPMI, centrifuged at 300 × g for 5 min at 4 °C, and the resulting pellets were snap-frozen and stored at −80 °C until RNA isolation.

### 2.4 RNA isolation and RNA library preparation and sequencing

Total RNA from HTR-8/SVneo cells treated with ethanol and/or _HEa_miRNAs mimics was isolated using the RNeasy Mini Kit (Qiagen, Cat. No. 74104), following the manufacturer’s protocol. RNA concentration was measured using Qubit RNA BR kit and were normalized to an equivalent starting concentration. RNA quality was assessed with the Agilent TapeStation 4200 RNA assay. All samples included in downstream analysis had RNA Integrity Number equivalent (RINe) values > 7. Libraries were prepared from 100 ng of total RNA per sample using the Illumina Stranded Total RNA Prep, Ligation with Ribo-Zero Plus kit (Illumina), which includes ribosomal RNA depletion. Samples were uniquely indexed and pooled for sequencing. Library quality was verified using the Agilent 2100 Bioanalyzer and DNA 1000 Kit. Paired end 150 bp sequencing was performed on an Illumina NextSeq 2000 using a P4 flow cell to generate ∼60 million reads per library. RNA sequencing data are available in NCBI GEO (*GSE324021*).

### 2.5 Quantification and RNA-sequencing analysis

Raw sequencing reads were assessed for quality using *FastQC* (Andrews, 2010) and trimmed for adapters and low-quality bases (Phred score <20) using *Trimmomatic* (Bolger et al., 2014). High-quality reads were mapped to the human reference genome (GRCh38/hg38) using *HISAT2* (Kim et al., 2015) with default parameters. Transcript-level counts were generated using *HTSeq* (Anders et al., 2015). All preprocessing steps, including quality filtering, trimming, alignment, and quantification, were performed using the Galaxy platform hosted by Texas A&M High Performance Research Computing (HPRC) facility (https://hprcgalaxy.tamu.edu/). Genes with fewer than 10 reads in ≥50% of samples were excluded. The remaining 18,387 genes were used for downstream analyses. Variance-stabilizing transformation (VST) was applied prior to visualization. Principal component analysis (PCA) and hierarchical clustering were used to assess sample quality and identify potential outliers. One sample was removed based on Cook’s distance and PCA separation. Differential expression analysis was conducted using the *DESeq2* package (Love et al., 2014) in the ‘*R’* programming environment. Genes with Benjamini-Hochberg adjusted p-values (FDR) < 0.05 and absolute log_2_ fold change ≥0.5 were considered significantly differentially expressed (differentially expressed genes, DEGs). This threshold, consistent with the published literature (St Laurent et al., 2013), captures moderate but biologically relevant, false-discovery rate-gated changes in gene expression, appropriate for pathway enrichment analysis, since miRNA-mediated regulation often results in subtle transcriptional shifts across large gene networks. All analyses included at least three biological replicates per group unless otherwise specified.

### 2.6 Pathway and network analyses

Hallmark gene sets associated with different cellular processes were extracted from the Molecular Signatures Database (MSigDB (Liberzon et al., 2015)). DEGs were categorized into six functional groups: metabolism, development (including EMT and angiogenesis), stress response, proliferation, immune response, and signaling pathways. Gene Set Variation Analysis (GSVA) was performed to evaluate pathway-level changes using the variance-stabilized expression matrix. GSVA calculates non-parametric enrichment score for each hallmark pathways, which provides measure of pathway activity. GSVA scores were then used to compare pathway-level differences across treatment groups. Weighted gene co-expression network analysis (WGCNA) was performed using variance-stabilized gene expression data on 17 samples (3 samples for each exposure group except 2 samples for _HEa_miRNAs mimics treatment and pre-ethanol exposure) to identify gene modules associated with treatment conditions and biological traits. Modules were assigned color codes, and hub genes were identified within each module based on intramodular connectivity. A topological overlap matrix (TOM) heatmap was generated to visualize network connectivity and module relationships. Module–trait correlations were assessed to link specific gene networks to ethanol exposure (pre-, post-, or no exposure) and _HEa_miRNAs mimics treatments. Pathway enrichment analysis was performed using the Kyoto Encyclopedia of Genes and Genomes (KEGG) database and Gene Ontology (GO) enrichment was performed for each module to identify overrepresented biological functions.

### 2.7 Statistical analyses

For cell viability and apoptosis assays, results were expressed as mean ± standard error of the mean (SEM). Group comparisons were conducted using one-way or two-way ANOVA, followed by Tukey’s *post hoc* test for multiple comparisons, as appropriate. Normality and variance assumptions were evaluated prior to parametric testing. Statistical analyses for these assays were performed using GraphPad Prism (version 10.0.2 (171)). A p-value <0.05 was considered statistically significant unless otherwise specified.

## 3 Results

### 3.1 Bulk RNA-Seq reveals miRNA-dependent transcriptomic signatures in ethanol-treated trophoblasts

We performed bulk RNA-seq on placental trophoblasts to examine the impact of sequential ethanol exposure and/or _HEa_miRNAs (microRNAs mimics) treatment. We used human trophoblast HTR-8/SVneo cells to investigate gene expression changes that could be relevant to placental and fetal development. Using Galaxy (V21.01) implemented on the institutional supercomputing platform (hprc.tamu.edu), raw reads were aligned to the Gencode GRCh38.v33 reference genome. A total of 60,616 genes were detected initially. Following stringent filtering criteria as described in the methods section, the dataset was refined to 39,017 unique transcripts. The expression data were normalized using size factor estimation. Our final dataset included 18,387 high-confidence, normalized genes for differential expression and network analyses.

**FIGURE 1:**
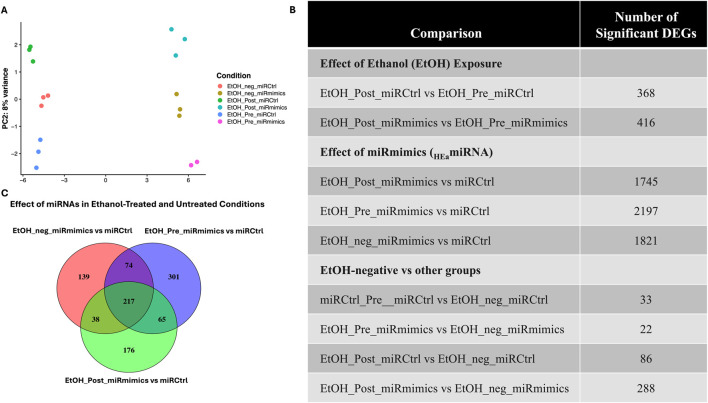
Differential Gene Expression Analysis. **(A)** Principal component analysis (PCA) was performed on the RNA-seq data, which shows the clustering of samples across six experimental groups. ‘EtOH_neg’ represents cells without ethanol exposure, ‘EtOH_pre’ indicates ethanol exposure before miRNA transfection, and ‘EtOH_Post’ indicates ethanol exposure after transfection in HTR8 cells. miRCtrl refers to scrambled control miRNA, and ‘miRmimics’ refers to _HEa_miRNAs treatment. We observed a clear separation of the samples by miRNA exposure on the 1st principal component, and ethanol exposure on the 2nd component. Each point represents an individual sample. **(B)** Number of DEGs identified in pairwise comparisons across treatment groups (at FDR <0.05, log2FC > 0.5). We assessed the effect of timing of ethanol (EtOH) exposure, _HEa_miRNA transfection, and their combined effects in HTR-8/SVneo cells. EtOH(neg) refers to PBS treated baseline controls. **(C)** Venn diagram showing the number of differentially expressed genes (DEGs) that are overlapped or uniquely altered by _HEa_miRNA in presence (pre- and post-exposure) and absence of ethanol (neg). Each group includes n = 2-3 biological replicates.

To assess the global transcriptomic changes induced by ethanol exposure and _HEa_miRNAs, we performed principal component analysis (PCA) on the normalized gene expression data. We observed one distinct outlier (a sample from the pre-ethanol exposure with _HEa_miRNAs mimics treatment group) that separated from all other samples in the initial PCA plot (Supplementary Figure S1A). Furthermore, Cook’s distance analysis also showed that this same sample had an abnormally high influence on the fitted model, which significantly exceeded the distribution of all other samples (Supplementary Figure S1B). Upon removal of the sample, clustering of the samples improved, with clear separation between experimental conditions (Figure 1A). In Figure 1A, principal components, especially PC1 captured the major sources of variation in the data. The samples clustered distinctly by treatment group, indicating separation of transcriptomic profiles due to _HEa_miRNA treatment on PC1 and ethanol exposure on PC2. The strong separation of transcriptomic profiles was observed between _HEa_miRNAs mimics and miRNA controls (explaining 80% of the variance on PC1), regardless of ethanol exposure, suggesting that _HEa_miRNA exposure was the major source of transcriptomic variation compared to ethanol (8% of the explained variance on PC2).

Analysis using the *DESeq2* package (methods) identified 6,976 significantly differentially expressed genes (DEGs; adjusted p-values (p_adj_ < 0.05) and a log2 fold-change threshold of 0.5). All pairwise comparisons between treatment groups resulted in significant DEGs (Figure 1B) using adjusted p-values (p_adj_ < 0.05) and a log2 fold change threshold of 0.5. We found a robust transcriptomic response elicited by both ethanol and miRNA treatment. Comparison between _HEa_miRNAs mimics and miRNA controls in pre- and post-as well as no-ethanol exposure resulted in the highest number of DEGs (2,197 DEGs, 1,745 DEGs and 1,821 DEGs respectively), indicating strong miRNA-mediated reprogramming of trophoblast transcriptomic signatures.

We then compared the DEGs across three different experimental conditions to understand the effect of _HEa_miRNAs in ethanol-treated and untreated conditions. We used three different experimental conditions and compared _HEa_miRNAs mimics vs. miRNA control in presence (both pre- and post-) and absence of ethanol. A Venn diagram showed both shared and condition-specific transcriptomic responses (Figure 1C). In total, 217 DEGs were commonly regulated across all three treatment conditions, which indicates that these genes represent a conserved _HEa_miRNA exposure signature regardless of ethanol exposure. However, we also found a substantial number of unique _HEa_miRNA-linked DEGs in each ethanol exposure condition. 139 DEGs were specific to the ethanol negative group, 301 DEGs uniquely regulated in pre-ethanol treatment groups, and 176 DEGs were unique to the post-ethanol treatment group. The substantial differences in the DEGs in pre- and post-ethanol treated groups highlights that timing of ethanol exposure significantly modulates the transcriptional effects of the _HEa_miRNAs mimics. The pre-ethanol exposure treated group showed the largest number of unique DEGs, suggesting early exposure during pregnancy may sensitize trophoblast cells to miRNA-induced transcriptional/epigenetic reprogramming.

### 3.2 Ethanol and _HEa_miRNA mimics alter hypoxia-associated responses and key placental gene expression

**FIGURE 2:**
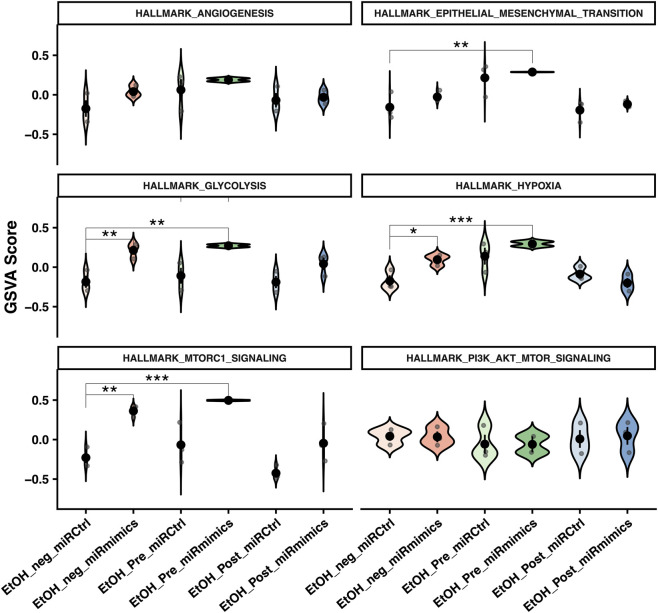
Gene Set Variation Analysis (GSVA) scores for selected hallmark pathways. Violin plots of group-wise variation in pathway activity showing significant difference across ethanol exposure and _HEa_miRNA treatment (*p < 0.05, **p < 0.01, ***p < 0.001). Each group includes n = 2-3 biological replicates.

To evaluate whether ethanol and _HEa_miRNA mimics exposures affect cellular pathways relevant to placental development, we performed Gene Set Variation Analysis (GSVA) using the Molecular Signatures Database (MSigDB) Hallmark gene sets collection (MSigDB-H, https://www.gsea-msigdb.org/gsea/msigdb/human/collections.jsp#H, (Liberzon et al., 2015). Among the significantly enriched pathways, we observed strong modulation of hallmark pathways such as Hypoxia, Glycolysis, Angiogenesis, Epithelial Mesenchymal Transition, and MTORC1 signaling, particularly in the pre-ethanol exposure and _HEa_miRNA mimic group (Supplementary Figure S2). The hypoxia gene set was significantly upregulated in the pre-ethanol exposed HTR8 cells which were subsequently transfected with _HEa_miRNA mimics, in comparison HTR8 cells that were not exposed to ethanol and transfected with scrambled, control miRNAs (Figure 2). The significant increase of hypoxia gene set was also observed with the _HEa_miRNA mimic treatment alone, i.e., in the absence of ethanol exposure, suggesting that _HEa_miRNAs alone could elevate the hypoxic response gene network, and that this effect was independent of ethanol exposure. Furthermore, pathway scores for glycolysis and mTORC1 signaling, which were both downstream of hypoxia-inducible pathways, were also elevated following _HEa_miRNA exposure, with or without ethanol pre-exposure, indicating a shift toward a glycolytic and metabolically active stress response to miRNAs.

**FIGURE 3:**
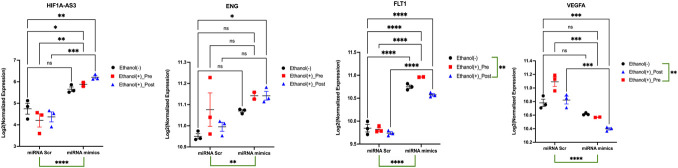
Expression of hypoxia and angiogenesis-related genes across treatment groups. Bar graphs show normalized expression (log2) of HIF1A-AS3, ENG, FLT1, and VEGFA in trophoblasts across ethanol exposure and miRNA mimic conditions. Error bars represent SEM. Statistical significance was assessed by two-way ANOVA with *post hoc* multiple-comparison testing. Black asterisks indicate significant post hoc comparisons between groups (*p < 0.05, **p < 0.01, ***p < 0.001, ****p < 0.0001). Green brackets indicate a significant main effect of miRNA treatment or ethanol exposure as determined by two-way ANOVA.

We performed further parametric analysis of key hypoxia-associated and placenta-relevant genes (Figure 3). A two-way ANOVA (_HEa_miRNA and ethanol as factors) revealed that HIF1A-AS3, a hypoxia-induced lncRNA linked to HIF1A regulation was significantly elevated by _HEa_miRNA mimics (main effect of _HEa_miRNA, F (1,11) = 72.04, p < 0.0001); as was ENG (Endoglin), the TGF-β co-receptor and preeclampsia biomarker (F (1, 11) = 12.58, p = 0.0046). Two critical regulators of angiogenesis, FLT1 and VEGFA, widely studied in hypoxic placenta (Palmer et al., 2017), were also investigated. mRNA for FLT1 (VEGFR1), which encodes both membrane-bound and soluble receptors that are elevated in hypoxic placenta (Li et al., 2005), was significantly increased in across the miRNA-mimic treated groups (two-way ANOVA: miRNA x ethanol interaction, F (2,11) = 5.5, p = 0.0219; main effect of miRNA mimics F (1,11) = 622.2, p < 0.0001; main effect of ethanol F (2,11) = 12.2, p = 0.0016). In contrast, transcript levels of the ligand, VEGFA, were reduced across the miRNA-mimic treated groups (two-way ANOVA: miRNA x ethanol interaction, F (2,11) = 7.75, p = 0.0080; main effect of miRNA-mimics F (1,11) = 92.99, p < 0.0001; main effect of ethanol F (2,11) = 10.61, p = 0.0027), indicating a strong miRNA-driven suppression of VEGFA that was further modulated by ethanol exposure. This opposing pattern of FLT1 and VEGFA regulation is consistent with a hypoxia-like, anti-angiogenic shift in trophoblast cells. These transcriptional changes suggest altered environment especially hypoxia related changes in response to ethanol and _HEa_miRNA mimics exposures that led to altered trophoblast function.

### 3.3 miRNAs mimics differentially regulate EMT, angiogenesis, and inflammation genes in ethanol-treated and untreated cells

**FIGURE 4:**
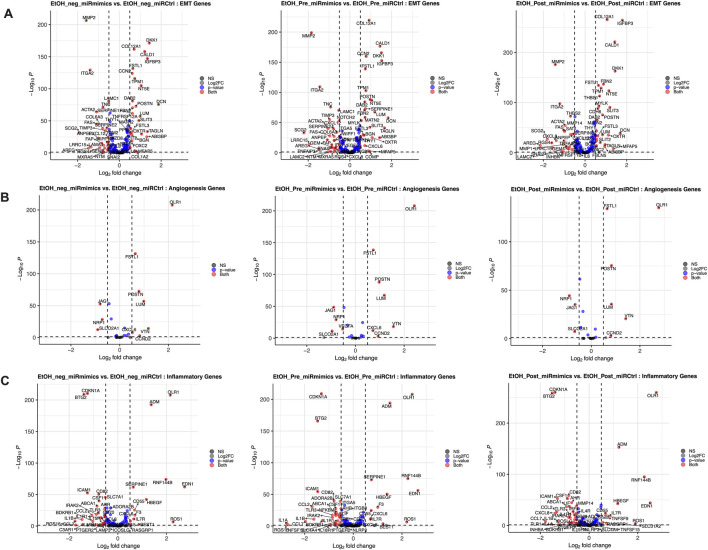
_HEa_miRNA-dependent regulation of EMT, angiogenesis, and inflammatory genes across ethanol exposure conditions. Volcano plots of log2 fold change and–log10 p-value of all genes differentially expressed in HTR-8/SVneo cells treated with _HEa_miRNA relative to miRCtrl under no ethanol, pre-ethanol, and post-ethanol exposure. **(A)** EMT-related genes, **(B)** angiogenesis-related genes, and **(C)** inflammatory genes are shown. The x-axis represents log_2_ fold change, and the y-axis represents–log_10_ adjusted p-value. Vertical dashed lines indicate fold-change thresholds, and the horizontal dashed line marks the significance cutoff. Highlighted genes were top 30 meeting both fold-change and significance criteria.

Volcano plot analysis identified up- and downregulated genes involved in epithelial-mesenchymal transition (EMT), angiogenesis, and inflammation in trophoblast cells in response to _HEa_miRNAs mimics treatment under different ethanol exposure conditions (Figure 4). Each of the gene sets includes comparison of miRNA mimics and miRNA controls in ethanol negative (left), pre-ethanol (middle), and post-ethanol (right) groups. EMT gene regulators such as MMP2, COL12A1, IGFBP3, and CALD1 were consistently upregulated in the _HEa_miRNAs mimic-treated trophoblast cells across all conditions (Figure 4A). A subset of angiogenic genes including OLR1, FSTL1, POSTN, and LUM were significantly upregulated and JAG1, NRP1, SLCO2A1 were significantly downregulated by _HEa_miRNAs mimics in all three conditions (Figure 4B). Several inflammation-related genes such as OLR1, ADM, EDN1, RNF144B were significantly upregulated and CDKN1A, BTG2, ICAM1, IL1B were downregulated with _HEa_miRNAs mimics treatment regardless of ethanol exposure (Figure 4C). The OLR1 gene transcript which encodes the receptor for oxidized low-density lipoprotein (LDL) was the most significantly upregulated mRNA, common to both the inflammatory and angiogenesis gene sets, whereas TGFB2 was the most significantly upregulated member of the apoptosis gene set. In each case, the significant regulation was due to _HEa_miRNA exposure, and ethanol exposure did not additionally, alter the effects of _HEa_miRNAs. The alteration in these different molecular signature gene sets highlights the dominant regulatory influence of _HEa_miRNAs mimics compared to the relatively modest additional effects of combined ethanol exposure.

### 3.4 Functional categorization reveals overlapping transcriptomic responses to ethanol and _HEa_miRNAs treatments

**FIGURE 5:**
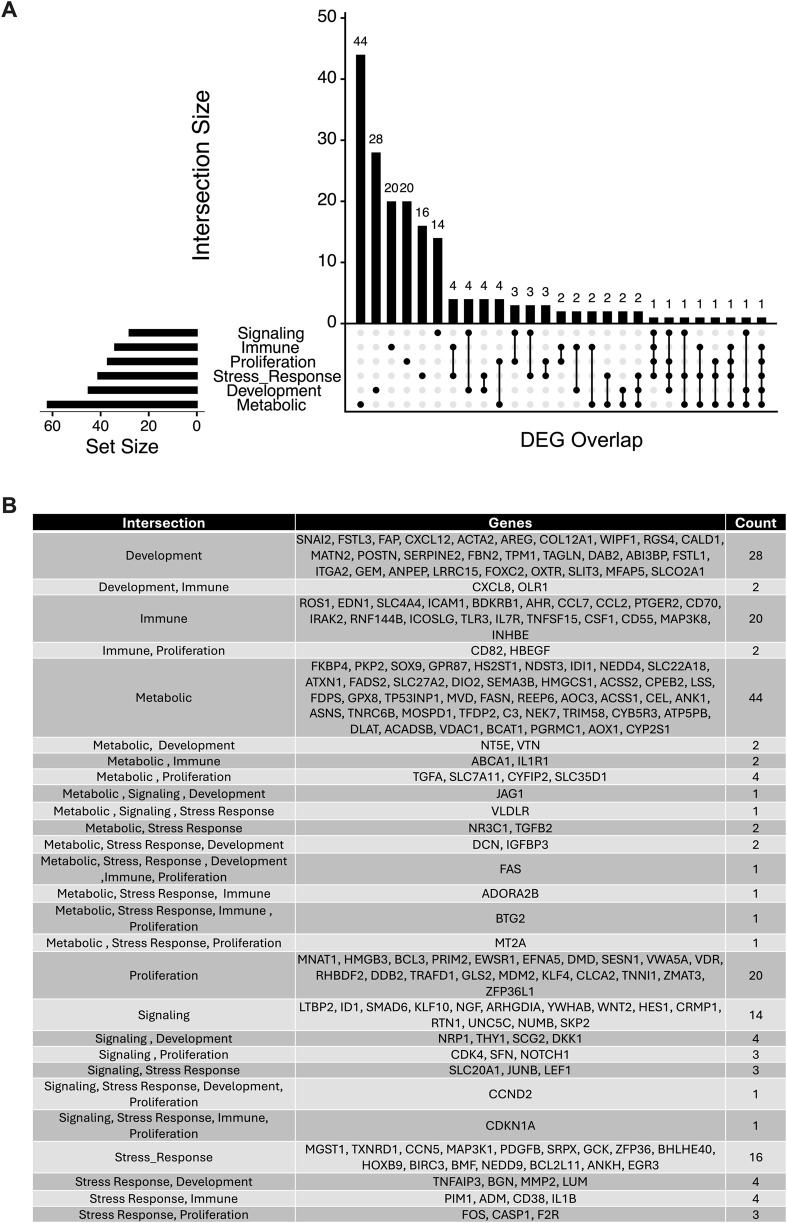
Functional categorization and overlap of DEGs across biological pathways. **(A)** DEGs identified across all comparisons were classified into six major functional categories: metabolism, development, stress response, proliferation, immune response, and signaling. *UpSet* plot summarizes the number of DEGs shared between one or more functional categories. The bar graph on the top indicates the number of overlapping genes in each intersection, while the matrix below shows which categories are involved in each intersection. The largest unique group consisted of 44 metabolism-related DEGs, followed by 28 genes involved in the development. The left bars show the total number of DEGs within each functional category. **(B)** Lists of genes by intersection type, providing the gene names and count per functional overlap. Several genes (e.g., FAS, CDKN1A, JAG1) appear in several categories, reflecting their multifunctional roles in trophoblast biology with ethanol and _HEa_miRNA treatment.

Both shared and condition-specific differentially expressed genes (DEGs) were identified by comparing _HEa_miRNA mimic-treated cells to miRNA control-treated cells within each of the following groups: ethanol-negative (no ethanol exposure), pre-ethanol (ethanol added before transfection), and post-ethanol (ethanol added after transfection), highlighting that ethanol exposure timing can influence the cellular response to _HEa_miRNAs. DEGs were next categorized into six major functional group (metabolic, development, stress response, proliferation, immune, and signaling) to better understand the biological relevance of these DEGs. The metabolic category included genes associated with glycolysis, bile acid metabolism, cholesterol homeostasis, fatty acid metabolism, heme metabolism, oxidative phosphorylation, and xenobiotic metabolism. Development-related genes were mapped to EMT and angiogenesis and stress response were mapped to reactive oxygen species (ROS), hypoxia, and apoptosis pathways. Proliferation-related genes were organized under G2/M checkpoint and p53 signaling pathways. Immune-related genes included inflammatory signaling and IL6/JAK/STAT3 pathways. The signaling category included genes from the TGF-β, PI3K/AKT/mTOR, Notch, Hedgehog, and Wnt/β-catenin signaling pathways. *UpSet* plot (Figure 5A) analysis includes the common DEGs shared across all three treatment groups, highlighting the transcriptomics effects of _HEa_miRNAs mimics outweighed and were independent of any effects of ethanol exposure. Metabolic-related DEGs were the largest (44 genes) amongst those functional categories followed by development-related (28), proliferation and immune-related genes (20), stress response-related (16), and signaling-related genes (14).

The *UpSet* plot also summarizes the number of DEGs overlap between one or more functional categories, highlighting their multifunctional biological roles in ethanol and miRNA treated trophoblast cells (Figure 5B). For instance, JAG1 was linked to metabolic, signaling, and developmental pathways; VLDLR was related to metabolic, signaling, and stress response; FAS was overlapped with five categories, including metabolic, stress response, development, immune, and proliferation; CDKN1A was associated with signaling, stress response, immune, and proliferation; CCND2 was involved in signaling, stress response, development, and proliferation; ADORA2B was related with metabolic, stress response, and immune regulation; BTG2 was related with metabolic, stress response, immune, and proliferation; and MT2A was involved with metabolic, stress response, and proliferation.

### 3.5 Weighted gene co-expression network analysis reveals treatment-specific transcriptomics modules

**FIGURE 6:**
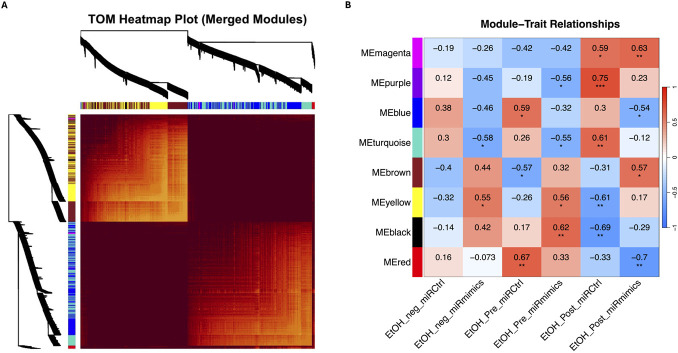
Weighted Gene Co-expression Network Analysis (WGCNA)- based transcriptomic profiling and module-trait relationships. **(A)** Topological overlap matrix (TOM) heatmap visualizing WGCNA within merged modules. Using hierarchical clustering and dynamic tree cutting, modules were identified and then they were merged based on module eigengene similarity. **(B)** Heatmap of WGCNA module correlations with the sample traits of ethanol exposure and _HEa_miRNA treatment. Each cell shows Pearson correlation (top) and p-value symbol (bottom) between module and sample traits. Red signifies positive associations and blue signifies negative associations. ME*blue*, ME*turquoise* were downregulated with _HEa_miRNA treatment regardless of the ethanol exposure and ME*brown*, and ME*yellow* were upregulated with _HEa_miRNA treatment. ME-module Eigengene.

To identify co-expressed trophoblast cells gene networks associated with _HEa_miRNAs mimics and ethanol exposure, we performed weighted gene co-expression network analysis (WGCNA). After merging similar clusters, we identified eight distinct gene modules, each labeled by unique color (Supplementary Figure S3). The topological overlap matrix (TOM) heatmap plot (Figure 6A) represents the network connection strength within and between modules, with clear clustering that supports the module structure. Each module represents a set of genes that are likely involved in related biological processes.

We examined the associations between each of these modules and sample traits (specifically, whether ethanol exposure occurred before or after miRNA transfection, and whether cells were treated with _HEa_miRNA mimics or scrambled controls) (Figure 6B). Timing of ethanol exposure (pre- and post-exposure, which is before and after the transfection of miRNAs) influenced gene network responses. Specifically, the *magenta*, *red*, *purple*, and *black* modules showed opposite correlation patterns between the pre- and post-ethanol groups. For instance, the *magenta* and purple modules were negatively correlated (r = −0.19 to - 0.56) with pre-ethanol exposure, while the red and black modules showed positive correlations (r = 0.17–0.67), suggesting that the gene networks involved in these modules respond differently depending on when the ethanol exposure occurred in relation to the miRNA treatment. The brown and yellow modules were positively correlated with _HEa_miRNA mimics but negatively correlated with _scr_miRNAs (miRNA controls). In contrast, the *blue* and turquoise modules showed an opposite pattern, suggesting a distinct gene expression shift driven by _HEa_miRNA mimics regardless of ethanol exposure. Minimal differences were observed between ethanol-exposed and unexposed conditions alone, suggesting that the gene network changes were primarily driven by _HEa_miRNA treatment and its interaction with the timing of ethanol exposure.

### 3.6 Functional analysis of modules highlights distinct gene programs responsive to _HEa_miRNA mimics

**FIGURE 7:**
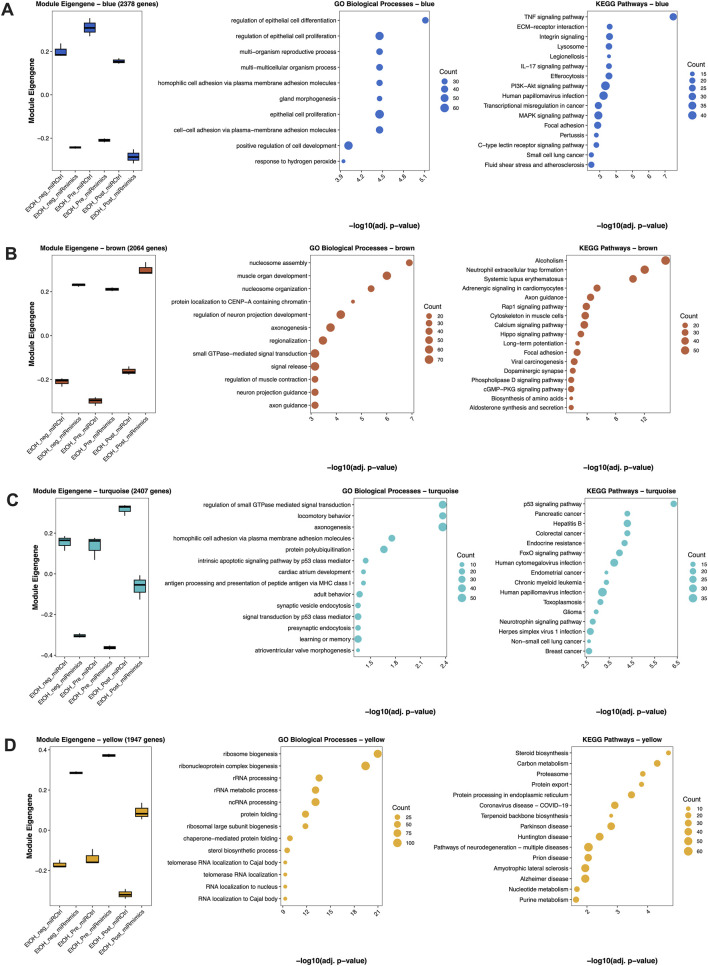
Weighted gene co-expression network analysis (WGCNA) identifies key transcriptional modules associated with _HEa_miRNA treatment in trophoblast cells. Four co-expression modules-blue **(A)**, brown **(B)**, turquoise **(C)**, and yellow **(D)** showed distinct eigengene expression patterns across experimental groups. Each panel shows three figures: left-module eigengene expression boxplots (y-axis) across experimental groups (x-axis); middle-enriched Gene Ontology (GO) biological processes for each module; right-enriched KEGG pathways based on the genes in each module. Dot size indicates gene count; x-axis shows log10 (adjusted p-value).

From the eight modules identified in our WGCNA, we selected four modules: the *blue*, *brown*, *turquoise*, and *yellow* modules for downstream analysis based on their distinct correlation patterns with _HEa_miRNA treatment and exposure timing and because they contained the largest number of genes, suggesting that they capture major transcriptional programs responsive to our treatments. The *blue* module (2,378 genes) was downregulated in response to _HEa_miRNA mimics regardless of ethanol exposure, when compared to _scr_miRNA-treated conditions (Figure 7A, left). Genes in this module were enriched for pathways involved in epithelial cell differentiation, proliferation, and adhesion (Figure 7A, middle panel) which are fundamental for trophoblast invasion and placental development. KEGG analysis showed genes involved in immune and inflammatory pathways such as IL-17, TNF, MAPK, PI3K-Akt, ECM-receptor interaction signaling (Figure 7A, right panel). The turquoise module's (2,407 genes) pattern of eigengene expression was similar to that of the *blue* module (Figure 7C, left). However, this module was enriched for small GTPase signaling, intrinsic apoptotic pathways, antigen presentation, behavior (Figure 7C, middle). KEGG pathways included p53, FoxO, and multiple cancer and infection-related pathways (Figure 7C, right), suggesting that turquoise module may be involved in cell signaling and stress-related transcriptional programs altered by _HEa_miRNA mimics treatment.

The *brown* module (2,064 genes) and the *yellow* module (1947 genes) were both highly expressed in cells treated with _HEa_miRNA mimics compared to those transfected with _scr_miRNA, indicating specific responsiveness to _HEa_miRNA exposure (Figures 7B,D, left). GO for biological process for the *brown* module showed chromatin assembly and developmental processes (Figure 7B, middle), whereas the *yellow* module was enriched for ribosome structure and biogenesis, and ncRNA processing (Figure 7D, middle). KEGG pathway analysis of *brown* module showed overrepresentation of genes associated with ‘alcoholism’, neutrophil extracellular trap formation, Rap1, calcium, and Hippo signaling, along with axon guidance and neurodevelopmental pathways (Figure 7B, right), whereas the *yellow* module included steroid biosynthesis, proteasome, and neurodegenerative disease pathways (Figure 7D, right). These four modules captured distinct transcriptional programs: *blue* (epithelial/immune), *brown* (chromatin/neurodevelopment), *turquoise* (apoptosis and cancer-related signaling), and *yellow* (protein synthesis and metabolic regulation).

### 3.7 Cytotoxic and apoptotic effects of ethanol on HTR-8/SVneo cells

To ensure that the transcriptomic changes observed were not induced by ethanol toxicity, we assessed the cytotoxic and apoptotic effect of ethanol and _HEa_miRNA in trophoblasts. We assessed the cytotoxic effect of ethanol on trophoblasts by measuring lactate dehydrogenase (LDH) as an indicator of cytotoxicity. HTR-8/SVneo cells were treated with 0, 60, 120, or 320 mg/dL ethanol for 24 or 48 h, with _HEa_miRNAs transfection included at 48 h time point. No significant increase in LDH activity was observed at either 24 or 48 h across all ethanol concentrations (Supplementary Figure S4A,B), as well as with or without _HEa_miRNAs (Supplementary Figure S3), suggesting minimal cytotoxicity under these conditions. Caspase-Glo® 3/7 assay was used to assessed apoptotic cell death analysis in trophoblasts. No significant changes were observed in the caspase activity across the ethanol concentration range (Supplementary Figure S4D). These results showed that the ethanol concentrations used for transcriptomic analyses (320 mg/dL) do not induce significant cytotoxicity, suggesting that the observed transcriptional changes reflect specific cellular responses to ethanol and _HEa_miRNAs exposure rather than nonspecific effects of cell death.

## 4 Discussion

Our earlier studies showed that delivery of synthetic murine-conserved _HEa_miRNAs caused placental and fetal growth restriction in mice (Tseng et al., 2019b) and transcriptomics analysis of the whole placenta pointed towards enrichment of Notch and EMT signaling (Pinson et al., 2023), suggesting a panel of microRNA mimics (_HEa_miRNAs) may have cell-type specific effects on placental development. In this study, we focused on extravillous human trophoblast cells (HTR-8/SVneo), which play an important role in placental invasion, spiral artery remodeling and maternal-fetal exchange. We investigated how exposure to high-dose ethanol (320 mg/dL) and _HEa_miRNAs, previously identified to induce altered birth outcomes and fetal development, modulate the transcriptomic landscape of these cells. Furthermore, this study allowed us to examine the individual and combined impact of ethanol and _HEa_miRNAs exposure on trophoblast function, and to identify specific gene networks that may contribute to placental dysfunction. Our findings suggest that _HEa_miRNAs alone have a significant global effect on the trophoblast transcriptome that is amplified, albeit to a smaller extent, by prior ethanol exposure. Collectively, our data suggest that PAE, in combination with elevated _HEa_miRNAs, may influence trophoblast gene expression programs relevant to placental function in our *in vitro* model, but these findings require further validation using *in vivo* models.

_HEa_miRNAs induced a strong and consistent effect on trophoblast gene expression. More than a thousand genes were significantly differentially expressed in response to _HEa_miRNA mimics in comparison to _scr_miRNA controls in both ethanol-treated (pre- and post-) and untreated cells (Figure 1B), and a large subset of these changes was shared across ethanol exposure conditions as shown in our PCA plot (Figure 1C). This conserved signature suggests that these _HEa_miRNAs have a core regulatory effect on trophoblast gene expression, independent of ethanol. These findings are consistent with prior studies showing that microRNAs can reprogram cellular transcriptional networks by targeting multiple mRNAs simultaneously (Bartel, 2018).

In this study, trophoblasts were treated with ethanol prior to, or following transfection with _HEa_miRNAs, to assess whether the sequence of exposure was important. The pre- and post-ethanol exposures serve to model different temporal windows of alcohol consumption. The pre-exposure model (prior to delivery of _HEa_miRNAs) was selected because of its face validity; a majority of pregnant women in our previous study curtailed their alcohol consumption following pregnancy awareness, though only some experienced elevated levels of _HEa_miRNAs. In contrast, the post-exposure paradigm models a smaller group that continued consumption through pregnancy. Pre-ethanol exposure followed by transfection with the pool of _HEa_miRNAs, the condition which most closely mimicked the chronology of our previous study in human populations, resulted in the largest number of unique DEGs (Figure 1B). The large number of DEGs in pre-ethanol treatment condition indicates that prior ethanol exposure may sensitize trophoblasts to subsequent miRNA-mediated transcriptional changes, and that timing of exposure is an important determinant of PAE outcomes. These findings support the hypothesis that alcohol reprograms gene expression in early placental development, which may compromise basal trophoblast functions and enhance the effect of _HEa_miRNAs. Such early reprogramming, as shown in the study that early alcohol exposure can alter epigenetic regulation and transcriptional programs in the placenta (Haycock, 2009), could explain the long-term placental dysfunction and fetal growth impairments in FASDs.

Gene enrichment variation analysis (GSVA) and targeted analysis of gene subsets highlighted specific patterns across the exposure groups (Supplementary Figure S2). Pre-ethanol exposed trophoblast cells transfected with _HEa_miRNA showed significant upregulation of MSigDB-H-curated hypoxia, glycolysis, mTORC1 pathways when compared to non-ethanol exposed, _scr_miRNA transfected cells (Figure 2). _HEa_miRNA treatment in the absence of ethanol also significantly upregulated the pathways when compared with _scr_miRNAs, suggesting the robust role of _HEa_miRNAs in the alteration of these pathways. These Hallmark-defined pathways significantly overrepresent hypoxia-associated signaling in treated trophoblasts, although they may also reflect a broader stress-adaptive response. While low oxygen tension is important for proliferation of cytotrophoblast during early placental development, persistent hypoxic signaling impairs the differentiation of cytotrophoblasts into invasive EVTs, developing a reduce capacity to remodel maternal spiral arteries (Caniggia et al., 2000; Maltepe and Fisher, 2015). Sustained or aberrant hypoxic signaling has been associated with placental pathologies such as Preeclampsia (PE) and Intrauterine Growth Restriction (IUGR) (Tal et al., 2010). In our study, the hypoxia-like state signature was accompanied by elevated transcript signatures for glycolysis, mTORC1 signaling, and downstream changes in angiogenic and apoptotic genes including increased FLT and endoglin (ENG), reduced VEGFA (Figure 3), which are known to be associated with pregnancy complications such as PE and IUGR (Levine et al., 2004). The opposing regulation of FLT1 and VEGFA suggests an anti-angiogenic shift, commonly observed under hypoxia-associated conditions, although similar patterns can also be observed as a component of cellular stress responses. These changes are in line with prior studies showing that overexpression of HIF-1α during pregnancy leads to elevation of soluble isoforms of FLT-1 and ENG, and to pregnancy complications (Tal et al., 2010). We also observed significant upregulation of HIF1a-AS3, a hypoxia-inducible long noncoding RNA, in _HEa_miRNA treated trophoblast cells, regardless of ethanol exposure. While HIF1a-AS3 has not been extensively studied in placenta, a recent study in ovarian cancer implicates its interaction with the RNA-binding protein YBX1 to regulate stress-adaptive gene expression, suggesting a role in gene regulation under stress conditions (Xie et al., 2023). Our results suggest that increased HIF1a-AS3 expression may also shape trophoblast responses under hypoxic stress environment. These transcriptional changes occurred even in the absence of cytotoxicity or apoptosis (as shown by LDH and Caspase-3/7 assays in Supplementary Figure S4) indicating that _HEa_miRNAs drive stress-adaptive gene programs rather than inducing direct cell injury.

We next examined whether _HEa_miRNAs modulate genes involved in epithelial-mesenchymal transition, angiogenesis, and inflammation, given their central role in trophoblast differentiation and placental development. Our results show that _HEa_miRNAs altered several EMT-related genes (ITGA2 and MMP2 downregulated, COL1A1 and COL12A1 upregulated) and angiogenesis-related genes (SLC02A1and JAG1 downregulated, FSTL1, CCND2, and OLR1 upregulated) regardless of ethanol exposure or the timing of treatment, indicating that _HEa_miRNAs together, have a dominant regulatory influence on these pathways in shaping core developmental gene programs. Inflammatory genes were more variable, with upregulation of OLR1, ADM, EDN1 and downregulation of CDKN1, BTG2, ICAM1, implying both stress and immune responses in the _HEa_miRNA treated trophoblasts. Importantly, our results showed the overlap of differentially expressed genes across various hallmark and functional categories. An *UpSet* plot-based approach (Figure 5) showed many DEGs contributed to more than one biological process, which highlights the complex, interconnected and pleiotropic transcriptomic effect of ethanol exposure and _HEa_miRNAs treatments. Notably, several genes like JAG1, FAS, CCND2, and CDKN1A were implicated in multiple functional categories including angiogenesis, apoptosis, immune and cell cycle regulation, and stress response highlighting their potential role as key regulatory hubs. The high degree of intersection among functional categories suggests that these transcriptomics changes in trophoblast cells are likely to entail coordination among a network of regulatory pathways, rather than a dominant contribution of any single pathway.

Out of eight modules that were identified from our WGCNA, the *blue, turquoise*, *brown* and *yellow* modules captured the core transcriptional programs altered in trophoblasts in response to _HEa_miRNA treatment regardless of ethanol exposure. The *blue* and *turquoise* modules were downregulated by _HEa_miRNA mimics and included genes involved in epithelial proliferation and differentiation, immune responses, and MAPK/PI3K signaling. These pathways are crucial for trophoblast invasion, migration, and immune crosstalk with the maternal environment (Knofler et al., 2019), suggesting that _HEa_miRNAs may diminish these key functions during early placental development. In contrast, the *brown* and *yellow* modules were upregulated by _HEa_miRNAs. KEGG pathway enrichment analysis indicated that the brown module was enriched for genes associated with the ‘alcoholism’. These include genes previously implicated in the pathogenesis of FASD and other placental dysfunctions, such as those associated with oxidative stress and epigenetic remodeling (Terracina et al., 2024), neutrophil extracellular trap formation, a marker for placental inflammation and immune dysregulation (Ramos et al., 2024), as well as Hippo, calcium, and Rap1 signaling, critical for syncytialization and placental barrier formation, and invasion (Gehl et al., 2025; Ray et al., 2022; Yoshie et al., 2023). The *yellow* module was enriched for ribosome biogenesis and proteostasis, suggesting that _HEa_miRNAs may control and dysregulate the high translational output needed in rapidly proliferating trophoblast cells. KEGG pathway enrichment analysis indicated that the *yellow* module was broadly, a metabolic function module (steroid biosynthesis, carbon metabolism, purine metabolism, and nucleotide metabolism). Steroid biosynthesis is particularly relevant in placental hormone production, and its dysregulation may impair endocrine functions of placenta. Furthermore, neurodegenerative disease (Alzheimer’s, Huntington’s, Parkinson’s, Amyotrophic Lateral Sclerosis) pathways enriched in the yellow module suggests cellular stress responses such as disrupted proteostasis, mitochondrial dysfunction, and altered protein turnover in trophoblasts due to _HEa_miRNA treatment. Together, these results suggest that _HEa_miRNA exposure, particularly in combination with ethanol, may disrupt trophoblast function, immune tolerance, and placental development, contributing to adverse pregnancy outcomes. Like the DEGs analysis and our volcano plots results, the WGCNA analysis highlighted that _HEa_miRNAs not only alter individual gene targets but reprograms entire gene networks that are central to placental development, immune modulation, and stress signaling.

This study has some limitations. For instance, HTR-8/SVneo cell lines, though extensively used to model placental pathologies, may not fully and accurately reflect the biology of primary extravillous trophoblasts because they were immortalized in the laboratory using the SV40 large-T-antigen (Graham et al., 1993), a process which may result in tumorigenic transformation (Ahuja et al., 2005). While our transcriptomic analyses showed that _HEa_miRNA altered the expression of a sets of genes linked to key biological processes that support placental growth and physiology, further validation is required when inferring functional outcomes. Importantly, our previous studies have demonstrated that _HEa_miRNAs regulate EMT markers at both the transcript and protein levels, impair trophoblast invasion in HTR-8 cells (Tseng et al., 2019b) and decrease murine placental and fetal growth *in vivo* (Pinson et al., 2023). Therefore, while the findings of the current study should be interpreted with caution and within the context of an *in vitro* model, and will need further validation, they are supported by our previous in both *in vivo* and *in vitro* models.

In summary, the reasons why the same levels of PAE can adversely affect some pregnancies but not others, are unclear. While socioeconomic and other factors may contribute to such variability, our previous findings that PAE pregnancies which resulted in adverse birth outcomes were also characterized by elevated _HEa_miRNAs in the 2nd trimester (Balaraman et al., 2016) provided evidence for one potential mechanistic biological explanation. This study extends the findings of that study by identifying _HEa_miRNAs rather than ethanol as the dominant driver of transcript level changes, at least in an invasive placental trophoblast cell subtype. Our RNA-seq and network analyses revealed that _HEa_miRNAs reprogram transcriptional networks that are critical for placental development and stress response, and their effects persist even in the absence of ethanol, suggesting that _HEa_miRNAs may act as dominant drivers of altered placental signaling in the context of prenatal alcohol exposure. We have found that prenatal alcohol exposure may synergize with regulatory RNAs to impair placental development, specifically the invasion, differentiation, proliferation, immune and stress response by trophoblast cells. Several hallmark processes such as hypoxia, EMT, angiogenesis, and inflammatory pathways were altered, which are known mediators of placental insufficiency, fetal growth restriction, and adverse pregnancy outcomes. In summary, these results support the hypothesis that _HEa_miRNAs function not just as biomarkers but as active endocrine mediators capable of modulating placental gene expression. These findings also point to potential for RNA-based or miRNA-targeted therapies to both preventative and therapeutic approach to improve placental function. However, these findings are based on an *in vitro* model and further understanding on how these miRNAs coordinate to shift multiple gene networks at once in the trophoblast may help identify new strategies for intervention in FASDs and pregnancy related outcomes. Future studies are needed to determine the long-term impact of these changes on placental function and fetal development, particularly *in vivo* models or primary placental tissues.

## Data Availability

The datasets presented in this study can be found in online repositories. The names of the repository/repositories and accession number(s) can be found below: https://www.ncbi.nlm.nih.gov/, GSE324021.

## Supplementary material

SUPPLEMENTARY FIGURE S1 Quality control and outlier detection. **(A)** PCA was performed on the RNA-seq data, which shows that the clear separation of the samples across treatment groups with one possible outlier in the EtOH(pre)_miRmimics (right pink point). Each point represents an individual samples. **(B)** Cook’s distance plot (log_2_ scaled) identifies the same sample (S5 from EtOH(pre)_miRmimics) from PCA plot has high leverage. Therefore, the sample (S5) was removed from downstream analysis.

SUPPLEMENTARY FIGURE S2 Distinct signatures observed by Gene Set Variation Analysis (GSVA) of hallmark pathways across treatments. Heatmap showing shifts in pathway activity across treatment groups using MSigDB Hallmark gene sets. Red implies higher activity, and blue implies lower activity relative to the dataset average.

SUPPLEMENTARY FIGURE S3 Hierarchical clustering gene dendrograms grouped into modules based on co-expression similarity. **(A)** Initial modules identified before merging. **(B)** Final modules after merging similar modules using height cut-off threshold of 0.15. Colors below the dendrograms indicate module membership.

SUPPLEMENTARY FIGURE S4 Assessment of cytotoxic and apoptotic effect of ethanol and _HEa_miRNA in HTR-8/SVneo trophoblasts. **(A,B)** LDH activity after 24 h (A) and 48 h (B) ethanol exposure (0–320 mg/dL), **(C)** LDH activity at 48 h in the presence or absence of ethanol and _HEa_miRNAs, **(D)** Caspase-3/7 activity at 48 h ethanol exposure (0–320 mg/dL). Statistical significance was assessed using one-way ANOVA with multiple comparisons; ns indicates non-significant differences (p > 0.05). Each dot represents an individual biological replicate.
