# Supplementary figures and images for: Maternal microRNAs linked to birth deficits due to prenatal alcohol exposure are dominant modifiers of gene expression in invasive trophoblast cells
Source: Front Cell Dev Biol. 2026 May 28;14:1834760. doi: 10.3389/fcell.2026.1834760 (PMC13254054; doi:10.3389/fcell.2026.1834760)

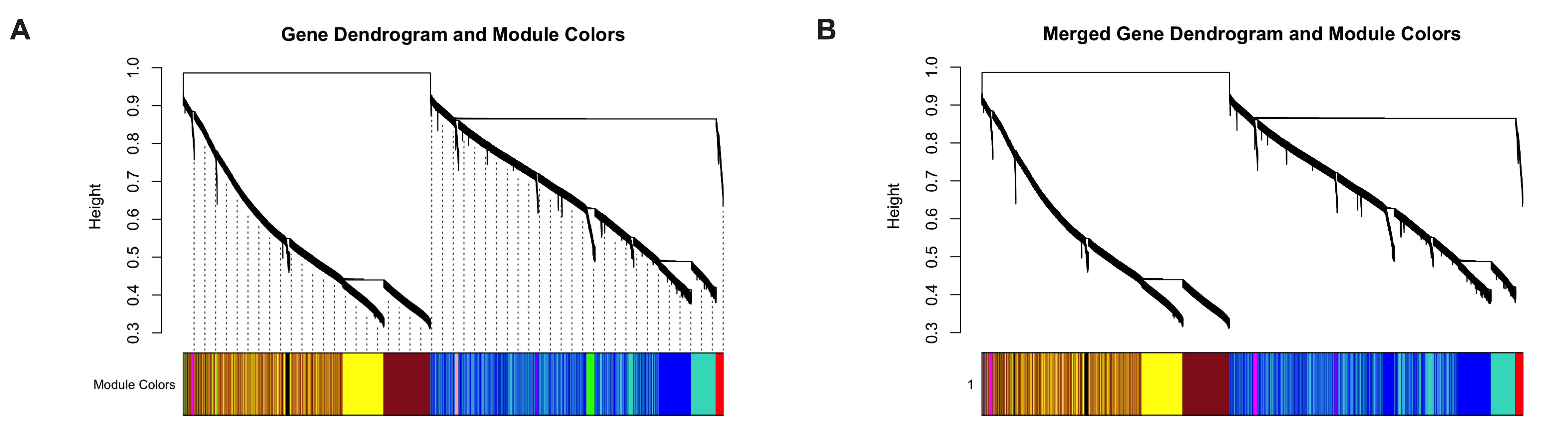

Supplement: Supplementary file 1 [file Image3.tiff]

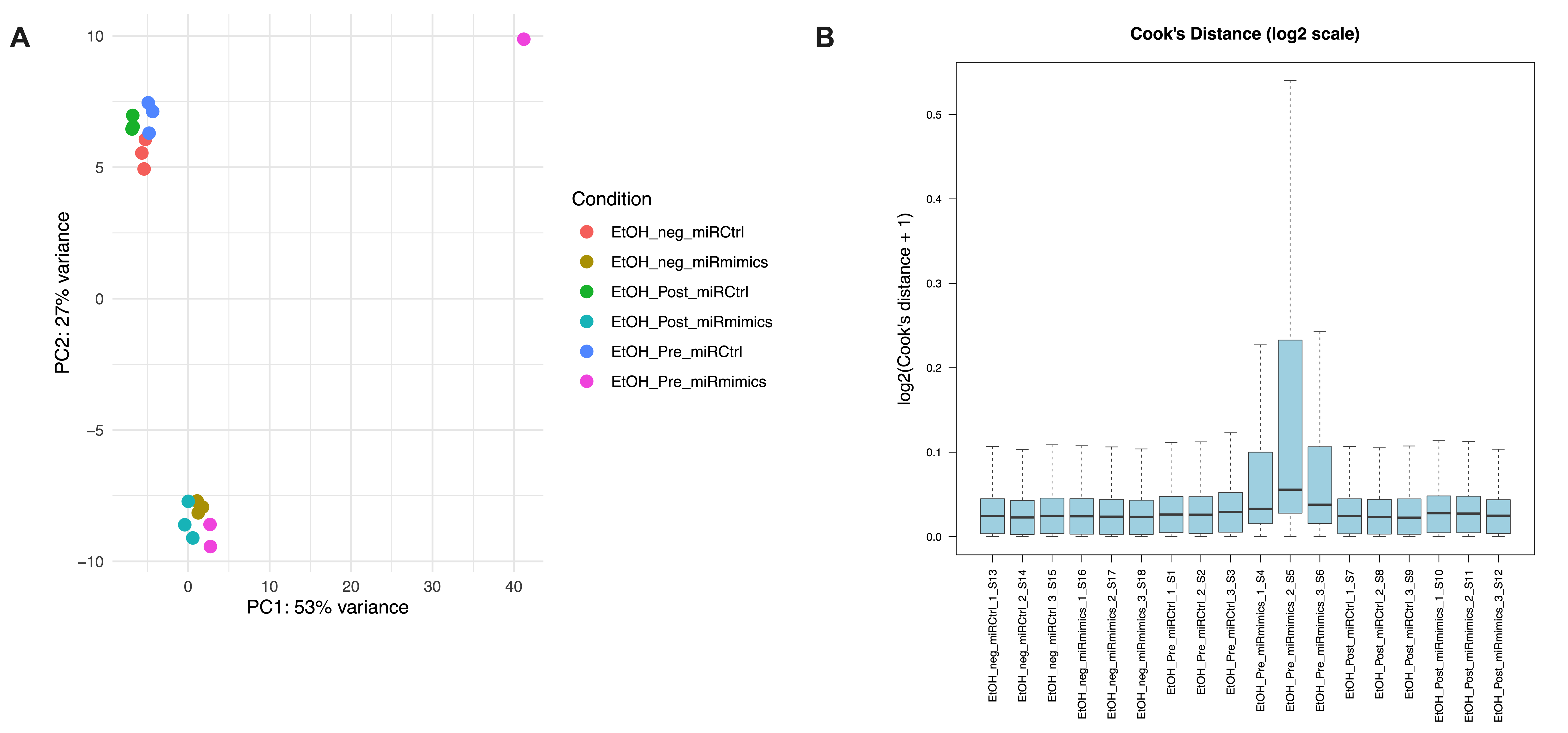

Supplement: Supplementary file 2 [file Image1.tiff]

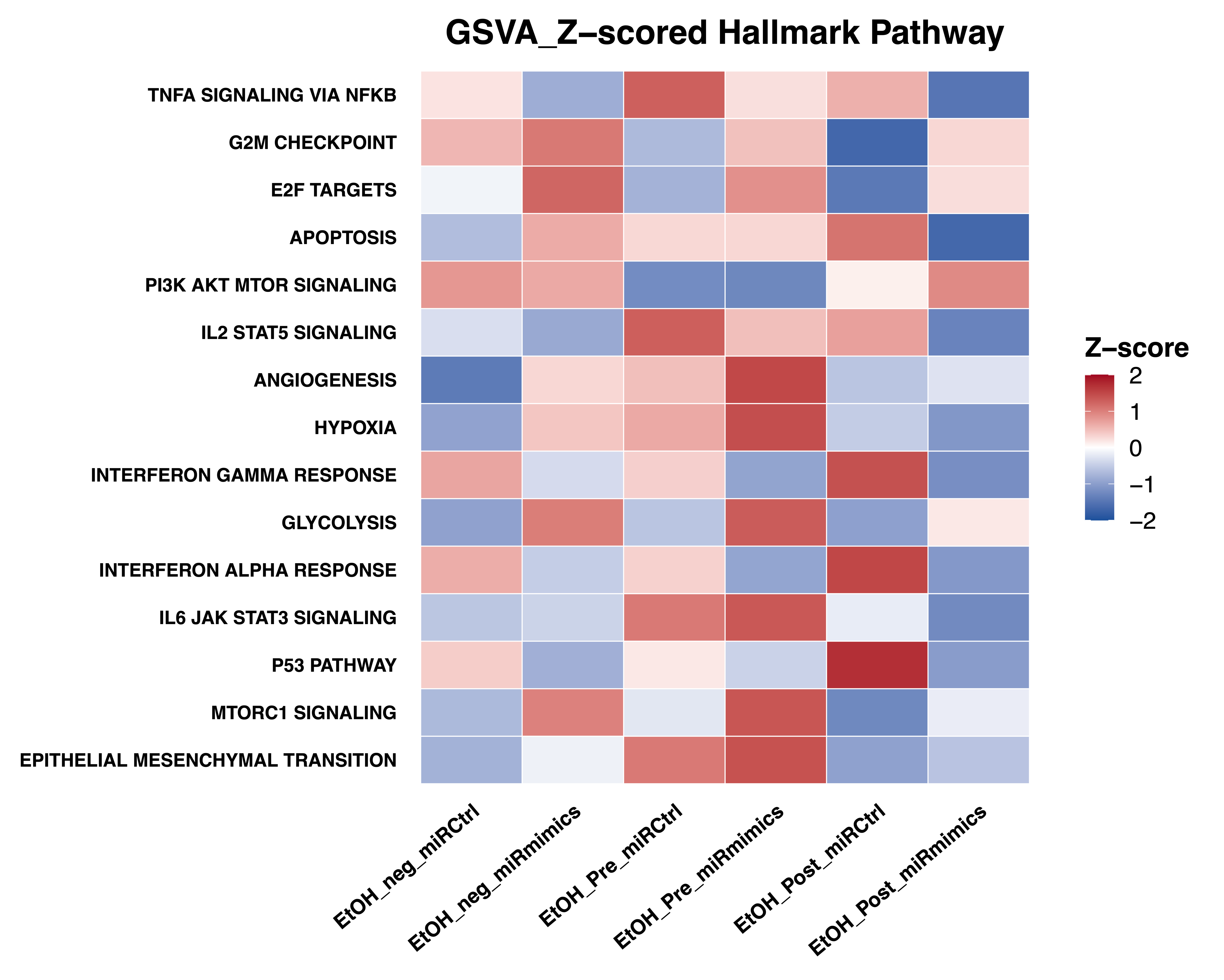

Supplement: Supplementary file 3 [file Image2.tiff]

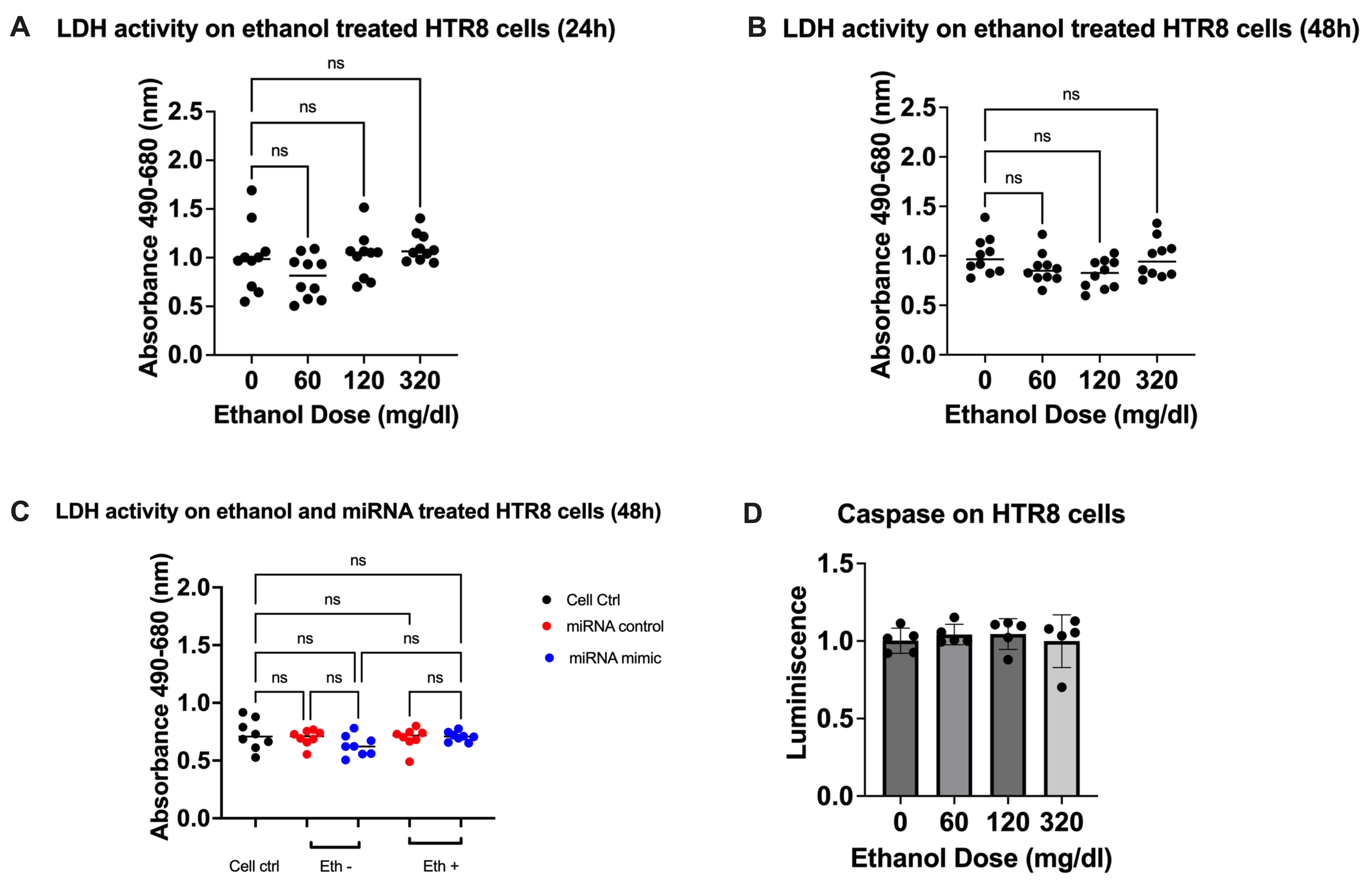

Supplement: Supplementary file 4 [file Image4.tiff]
